# Effect of Nebulized Amphotericin B in Critically ill Patients With Respiratory *Candida* spp. De-colonization: A Retrospective Analysis

**DOI:** 10.3389/fmed.2021.723904

**Published:** 2021-09-03

**Authors:** Hangxiang Du, Limin Wei, Wenzhe Li, Bixia Huang, Yongan Liu, Xiaofei Ye, Sheng Zhang, Tao Wang, Yizhu Chen, Dechang Chen, Jiao Liu

**Affiliations:** ^1^Department of Intensive Care Medicine, Ruijin Hospital Affiliated to Shanghai Jiaotong University School of Medicine, Shanghai, China; ^2^Department of Women Health Care, Maternal and Children Healthcare Hospital of Jiading District, Shanghai, China; ^3^Department of Intensive Care Medicine, Xinchang Hospital of Traditional Chinese Medicine, Shaoxing, China; ^4^Department of Health Statistics, Second Military Medical University, Shanghai, China

**Keywords:** amphotericin B, mechanical ventilation, *Candida* spp. colonization, ventilator-associated pneumonia, Candida score

## Abstract

The potential relationship among airway *Candida* spp. de-colonization, nebulized amphotericin B (NAB), and occurrence of ventilator-associated pneumonia (VAP) in patients who are critically ill has not been fully investigated, especially concerning effects on survival. In this observational, retrospective, cohort study in a 22-bed central intensive care unit, we included patients aged >18 years who required mechanical ventilation (MV) for >48 h, with at least two consecutive positive *Candida* spp. test results. Patients were categorized into NAB and no NAB (control) groups. Propensity matching at 1:1 was performed according to strict standards, and multiple Cox proportional hazard model and multivariate analyses were performed to evaluate the effects of NAB treatment. Throughout an 8-year study period, 526 patients had received MV and had positive respiratory tract *Candida* spp. cultures. Of these, we included 275 patients and excluded 251 patients. In total, we successfully matched 110 patients from the two groups (each group, *n* = 55; total population median age, 64 years; Acute Physiology and Chronic Health Evaluation II [APACHE II] score, 25.5; sequential organ failure assessment score, 9). The *Candida* spp. de-colonization rate was 69.1% in patients treated with NAB. VAP incidence did not differ significantly between the NAB (10.91%) and control (16.36%) groups (*P* = 0.405). *Pseudomonas aeruginosa-*related VAP rates differed significantly between the NAB (10.91%) and control (25.45%) groups (*P* = 0.048). Five (9.1%) patients in the NAB group died during hospitalization compared with 17 (30.9%) controls (*P* = 0.014). At 28 days, 9 (16.4%) and 16 (29.1%) deaths occurred in the NAB and control groups, respectively, (*P* = 0.088). The cumulative 90-day mortality rate differed significantly between the two groups (23.6 vs. 43.6%, *P* = 0.015). Multivariate logistic regression analyses indicated a decreased 90-day mortality in the NAB group (adjusted odds ratio 0.413; 95% confidence interval 0.210–0.812; *P* = 0.01). In subgroup analyses, the NAB-associated decreased risk of death at 90 days was consistent across subgroups of patients with a *Candida* score of 2, younger age (<64 years), a higher APACHE II score (≥25), fewer *Candida* sites (<2), or MV at admission. NAB treatment contributed to *Candida* spp. airway de-colonization, was associated with a reduced risk of *P. aeruginosa-*related VAP, and improved 90-day mortality in patients critically ill with *Candida* spp. tracheobronchial colonization who had received MV for >2 days. NAB may be an alternative treatment option for critically ill patients with VAP.

## CANDIDA

*Candida* spp. are commonly found in patients in intensive care units (ICUs) who are mechanically ventilated, occurring in approximately 30% of patients receiving mechanical ventilation (MV) for >48 h and in 50% of those with a clinical suspicion of ventilator-associated pneumonia (VAP) (1). VAP is the most common hospital-acquired infection in ICUs, with a high incidence of 23.72/1,000 ventilator days (2). The association among VAP, increased morbidity and mortality rates, and additional healthcare costs has previously been reported (3, 4).

However, determining the clinical significance of *Candida* spp. colonization in the respiratory tract is challenging. When *Candida* spp. are isolated from the respiratory tract, discrimination between relatively harmless colonization and invasive infection remains challenging and has led to therapeutic dilemmas (5, 6). Fungal airway colonization is a frequent finding in patients undergoing MV (7). Some retrospective studies have shown that the presence of *Candida* within the airway cannot be considered harmless, and higher mortality rates have been reported when respiratory secretions have been *Candida* spp.-positive. Several clinical data suggest that *Candida* spp. airway colonization plays a significant role in the development of bacterial pneumonia. In a cohort study, Azoulay et al. reported that *Candida*bronchial colonization of *Candida* spp. increased the risk of *Pseudomonas aeruginosa-*related pneumonia (8); however, they did not consider the appropriateness of first-line antibiotic treatment. Furthermore, airway secretion cultures in those studies were not performed on specific fungal media, which could have led to an underestimation of yeast colonization (9). More recently, *Candida* spp. airway colonization was found to be independently associated with *Acinetobacter baumanii*-related VAP (10).

In addition to recent experimental data, these findings suggest that bacteria could take advantage of the presence of *Candida albicans* within the airway of patients undergoing MV (11). We hypothesized that the most resistant species could be selected and that the temporal relationship between *Candida* airway *Candida*colonization and the subsequent incidence of bacterial VAP was complex but that a patient's condition may improve with elimination of *Candida* spp. from the airway. However, this aspect has rarely been investigated.

Along with being routinely used intravenously, nebulised amphotericin B (NAB) has been used to eradicate *Candida* spp. from the lower respiratory tract as part of de-contamination protocols in patients with persistent *Candida* spp. colonization (12). During inhalation, high drug concentrations can reach deep alveoli while reducing nephrotoxicity and drug interactions. As such, NAB is both a useful and non-invasive treatment method. However, given the lack of effective data and standard methods for nebulized administration, inhalation of amphotericin B has been restricted to off-label use only.

To the best of our knowledge, for patients who are critically ill, the potential relationship between airway *Candida* spp. de-colonization with NAB and the incidence of VAP has not been adequately investigated, especially in terms of effects on survival. In this context, we aimed to undertake a retrospective study to assess the role of *Candida* spp. de-colonization with NAB in the development of subsequent bacterial VAP and to determine the effect on survival in patients who had undergone MV for >2 days.

## Methods

### Patient Selection

In this observational, retrospective, cohort study, which was conducted in a 22-bed central ICU in Shanghai Ruijin Hospital, China, we included patients who had been admitted between 1 December, 2012 and 31 December, 2020.

We retrospectively collected data from our patient data management system, according to a predefined checklist. We included all patients aged >18 and <85 years who required MV for >48 h, with at least two consecutive daily positive *Candida* spp. cultures taken from the lower respiratory tract (tracheal aspirate or bronchoalveolar lavage) as confirmation of respiratory colonization.

We excluded patients with immunodeficiency (human immunodeficiency virus infection, neutropenia [leucocyte count, below 0.5 × 10^9^/L]), active malignancy, a history of solid organ or bone marrow transplantation, long-term [>3 months] or high-dose [>1 mg/kg] steroid use or the use of other immunosuppressant medications, and those who had VAP prior to NAB treatment. Patients will be excluded if they received intravenous antifungal therapy during hospitalization.

This study was approved by the Medicine Institutional Review Board of Shanghai Ruijin Hospital.

### Definitions

For the diagnosis of VAP, we applied the American Centers for Disease Control and Prevention definition (13), which is based on a combination of radiographic evidence, clinical signs, and microbiological criteria. More specifically, radiographic evidence comprised two or more serial chest plain radiographs that included at least one of the following findings: new or progressive and persistent infiltrate(s), consolidation, or cavitation. Clinical and/or laboratory signs were as follows: temperature (>38 °C), leucopoenia (<4,000 white blood cells/mm^3^) or leukocytosis (≥12,000 white blood cells/mm^3^), and new-onset purulent sputum/cough/PaO_2_ decline/rales or bronchial breath sounds. Microbiological criteria were used to determine the causative organisms. Only bacteria whose concentration fulfilled at least one of the following criteria were considered VAP pathogens: protected specimen brush, ≥10^3^ colony forming unit (CFU)/mL; bronchoalveolar lavage fluid, ≥10^4^ CFU/mL; and endotracheal aspirate, ≥10^5^ CFU/mL.

Colonization was defined as the presence of *Candida* spp. in two or more consecutive bronchoalveolar lavage or sputum samples obtained on different days in the ICU, exclusive of infection, and the colonization start date was defined as the first positive sample. De-colonization was defined as the absence of *Candida* spp. in two consecutive samples on different days or the absence of *Candida* spp. in the last available sample.

### Data Collection

We retrospectively reviewed patient electronic medical records for the following information: demographic characteristics (age and sex), severity of illness (measured using the Acute Physiology and Chronic Health Evaluation [APACHE] II score and Sequential Organ Failure Assessment [SOFA] score on the day of ICU admission); admission diagnosis (cardiac, respiratory, or neurologic failure, infection); reason for admission to the ICU (cardiac or respiratory failure, coma); comorbidities; central venous catheterization; drug treatment (vasopressors, antibiotics); duration of MV; *Candida* airway colonization and species; in-hospital mortality; duration of hospitalization and ICU stay; and development of VAP with its microbiological etiology.

### Study Endpoints

The primary endpoint of the study was the 90-day mortality rate. The second endpoint included the *Candida* de-colonization rate, the incidence of VAP, the duration of MV, and the length of hospital and/or ICU stay.

### Statistical Analysis

Patients were categorized into two groups, namely, those with tracheobronchial colonization and treated with NAB (NAB group) and those with tracheobronchial colonization but no NAB (control group). Propensity score matching analysis (NAB vs. no NAB) was done (nearest neighbor, 1:1, matching with no replacement, caliper 0.02). Each patient was matched to a control according to the following criteria: age, severity of illness at ICU admission (PaO_2_/FiO_2_ ratio, SOFA and APACHE II scores, and the *Candida* score), comorbidity, and laboratory examination at admission (white blood cell count, procalcitonin, and C-reactive protein levels).

Categorical variables are presented as numbers and percentages and were compared using chi-square tests, whereas continuous variables are presented as means with standard deviations and were compared using one-way analysis of variance. Mann–Whitney U and chi-square tests were performed to compare continuous variables and categorical variables, respectively.

Biomarkers are described as medians with quartiles and were compared using a Kruskal–Wallis test and Kaplan–Meier curves were constructed. Log rank testing was performed to evaluate group differences in colonization and ventilation duration.

We calculated the crude odds ratio (OR) and the adjusted OR in relation to matching criteria and unbalanced variables. Analyses were adjusted for patient characteristics (age, APACHE II and SOFA scores, presence/absence of any chronic illness at ICU admission).

To determine the effect of NAB treatment on 28- and 90-day mortality, we conducted multiple Cox proportional hazard model, multivariate, and subgroup analyses. To minimize or avoid immortal time bias, we used landmark analysis and set the cut-off time point at the 9th day (the median time to receive NAB) after enrollment. To achieve this, we only select the patients who survived longer than 9 day and compared the survival time between the control group and NAB group. Hazard ratios (HR) and 95% confidence intervals (CI) were calculated. A *P* < 0.05 was considered statistically significant for all analyses. An SAS (version 9.1; SAS Institute, Cary, NC, USA) statistical software package was used for all statistical analyses.

## Results

### Study Patient Characteristics

**Figure 1:**
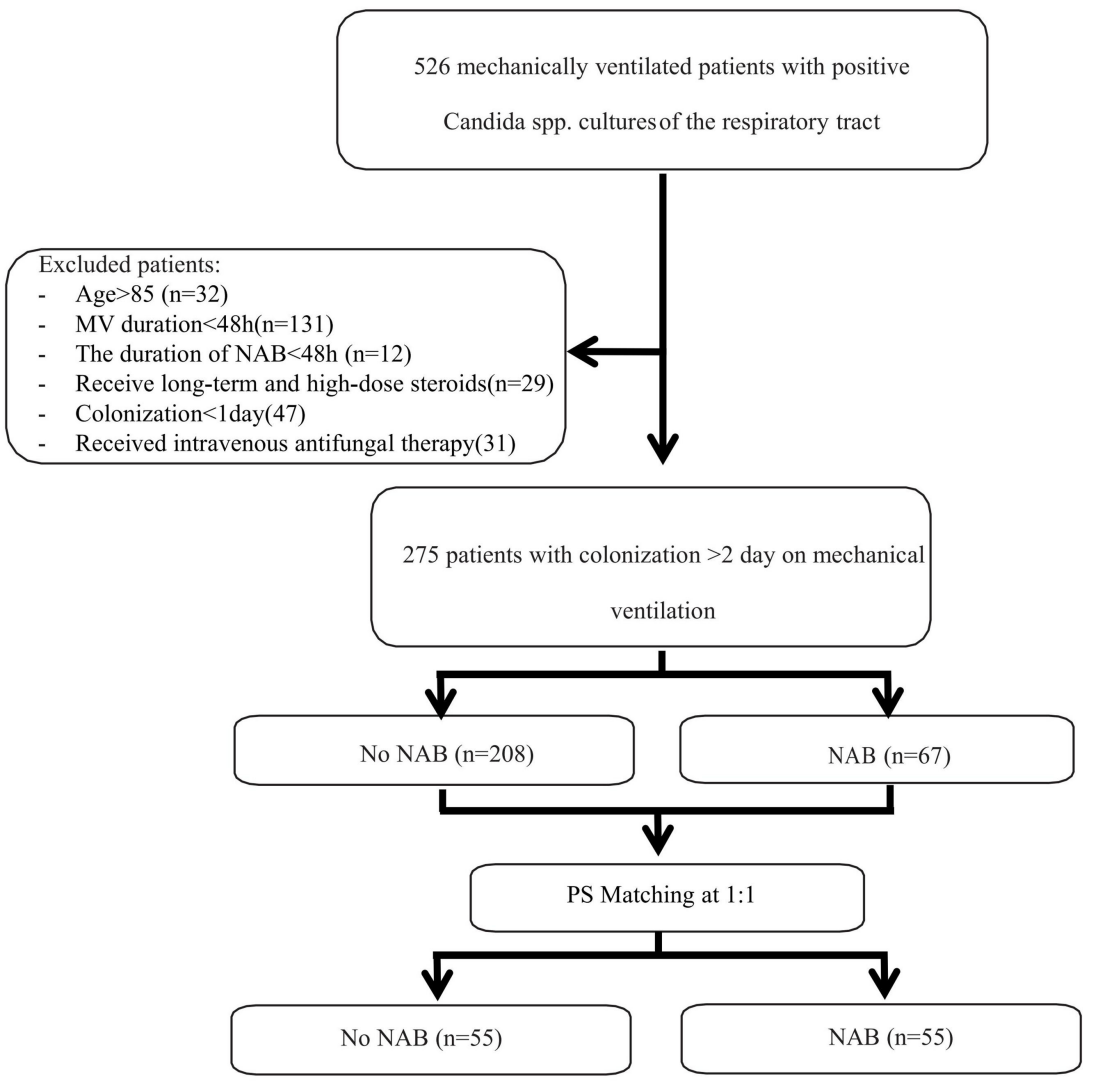
Flow diagram. NAB, Nebulized amphotericin-B.

During the 8-year study period, 526 patients received MV and were found to have positive *Candida* spp. cultures of the respiratory tract. Of these, 275 patients were eligible for inclusion in the study and 251 patients were excluded (please see Figure 1). The characteristics of patients who received NAB (*n* = 67) and those who did not (*n* = 208) before adjusting are summarized in Table 1. In the NAB group, the median age was 65 (interquartile range [IQR], 54–74) years, 53 (79.1%) patients were male, and 14 (20.9%) patients were female. In the control group, the median age was 64 (IQR, 50–74) years, 152 (73.1%) were male, and the *P*-value was not statistically significant.

**Table 1:** Patient baseline characteristics before adjustment.

**Variables**	**NAB group (*n* = 67)**	**No NAB group (*n* = 208)**	***P*-value**
Age(yr), median(IQR)	65 (54,74)	64 (50,72)	0.550
Sex, male, n%	53 (79.1)	152 (73.1)	0.325
Any comorbidity, n (%)			
Chronic obstructive pulmonary disease, n (%)	5 (7.46)	6 (2.88)	0.192
Diabetes	16 (23.88)	27 (12.98)	0.033
Hypertension	30 (44.78)	82 (39.42)	0.438
Chronic cardiac disease	15 (22.39)	29 (13.94)	0.101
Chronic kidney disease	15 (22.39)	17 (8.17)	0.002
Chronic liver disease	1(1.49)	5(2.40)	1.000
Stroke	14 (20.90)	20 (9.62)	0.015
Cause for ICU admission, n (%)			<0.0001
Postoperative care	4 (5.97)	33 (15.87)	
Pneumonia			
HAP	15 (22.39)	2 (13.94)	
CAP	8 (11.94)	3 (1.44)	
AECOPD	0 (0.00)	1 (0.48)	
Septic shock	8 (11.94)	9 (4.33)	
Congestive heart failure	4 (5.97)	10 (4.81)	
Trauma	9 (13.43)	62(29.81)	
Nerve system disease	15 (22.39)	55 (26.44)	
Post-CPR	3 (4.48)	2 (0.96)	
Others	1 (1.49)	4 (1.92)	
Signs and symptoms at admission			
Fever, n (%)	30 (44.78)	134 (64.42)	0.004
Cyanosis, n (%)	1 1.5)	15 (7.2)	0.082
Highest temperature (°C), median (IQR)	37.2 (36.8, 38)	37.6 (37,38.3)	0.009
Systolic pressure (mmHg), median (IQR)	127 (100,155)	132 (115, 151)	0.223
Diastolic pressure (mmHg), median (IQR)	76 (60, 85)	74 (63, 86)	0.557
Heart rate (bpm), median (IQR)	102 (83, 120)	94(80, 109)	0.021
Respiratory rate (bpm), median (IQR)	19 (16, 23)	17 (15, 21)	0.007
Rhonchus, n (%)	3 (4.5)	12 (5. 8)	0.917
Moist rales, n (%)	17 (25. 4)	38 (18.3)	0.206
PaO2/FiO2 at admission, median (IQR)	196 (134, 262)	279 (172, 358)	<0.001
SOFA Score at admission, median (IQR)	9 (8, 12)	7 (6, 9)	<0.001
APACHE II Score at admission, median (IQR)	28 (21, 32)	20 (15, 25)	<0.001
Respiratory Support at admission, n (%)			0.744
Nasal cannula	10 (15.15)	35 (16.83)	
High-flow nasal cannula	4 (6.06)	3 (1.44)	
Noninvasive mechanical ventilation	1 (1.52)	1 (0.48)	
Invasive mechanical ventilation	51 (77.27)	169 (81.25)	
Physiologic parameters at admission			
WBC(×10^9^/L), median (IQR)	10.11 (7.14, 14.73)	11.21 (8.32, 14.63)	0.140
Hemoglobin (g/L), median (IQR)	101 (81, 114)	106 (91, 123)	0.027
Platelets (×10^9^/L), median (IQR)	154 (109, 213)	141.5 (102, 199)	0.301
Lymphocytes (×10^9^/L), median (IQR)	0.67 (0.38, 1.03)	0.62 (0.44, 0.93)	0.669
Neutrophile granulocyte (×10^9^/L), median (IQR)	8.46 (5.7, 13)	9.82 (6.88 13.2)	0.097
N/L Ratio, median (IQR)	13.43(6.28,24.67)	16.21(9.44,23.46)	0.248
PT(seconds), median (IQR)	13.6 (12.3, 16.1)	12.9 (11.8, 14.5)	0.015
D-dimer (μg/mL), median (IQR)	1.96 (0.72, 4.45)	1.65 (0.66, 4.77)	0.910
TBil (μmol/L), median (IQR)	15.1 (9.2, 28.5)	14.3 (10.7, 21.85)	0.597
Scr (μmol/L), median (IQR)	91 (61, 167)	79.5 (64, 113)	0.095
Procalcitonin (ng/mL), median (IQR)	0.74 (0.3, 2.76)	0.53 (0.17, 2.44)	0.102
CRP (mg/L), median (IQR)	72 (42, 144)	29.5 (10, 86)	<0.001

*PT, partial thromboplastin time; TBil, total bilirubin; Scr, serum creatinine; CRP, C-reactive protein*.

The median APACHE II score was 28 (range, 21–32) and 20 (range, 15–25) in the patient and control groups, respectively. The median PaO_2_/FiO_2_ was 196 (IQR, 134–262) at ICU admission in the NAB group, which was lower than that in the control group (279; IQR, 172–358), with a significant difference (*P* < 0.001). There were significant differences in baseline characteristics between the two groups with respect to APACHE II and SOFA scores, admission diagnoses, and the PaO_2_/FiO_2_ ratio.

Patients in the NAB group were admitted mostly for respiratory diseases, and they had a higher fever, heart rate, and respiratory rate than those in the control group at ICU admission. No statistically significant differences between the two groups were observed concerning white blood cell count; platelet, lymphocyte, neutrophil granulocyte, D-dimer, total bilirubin, serum creatinine, and procalcitonin levels; or neutrophil-to-lymphocyte ratio at ICU admission.

**Figure 2:**
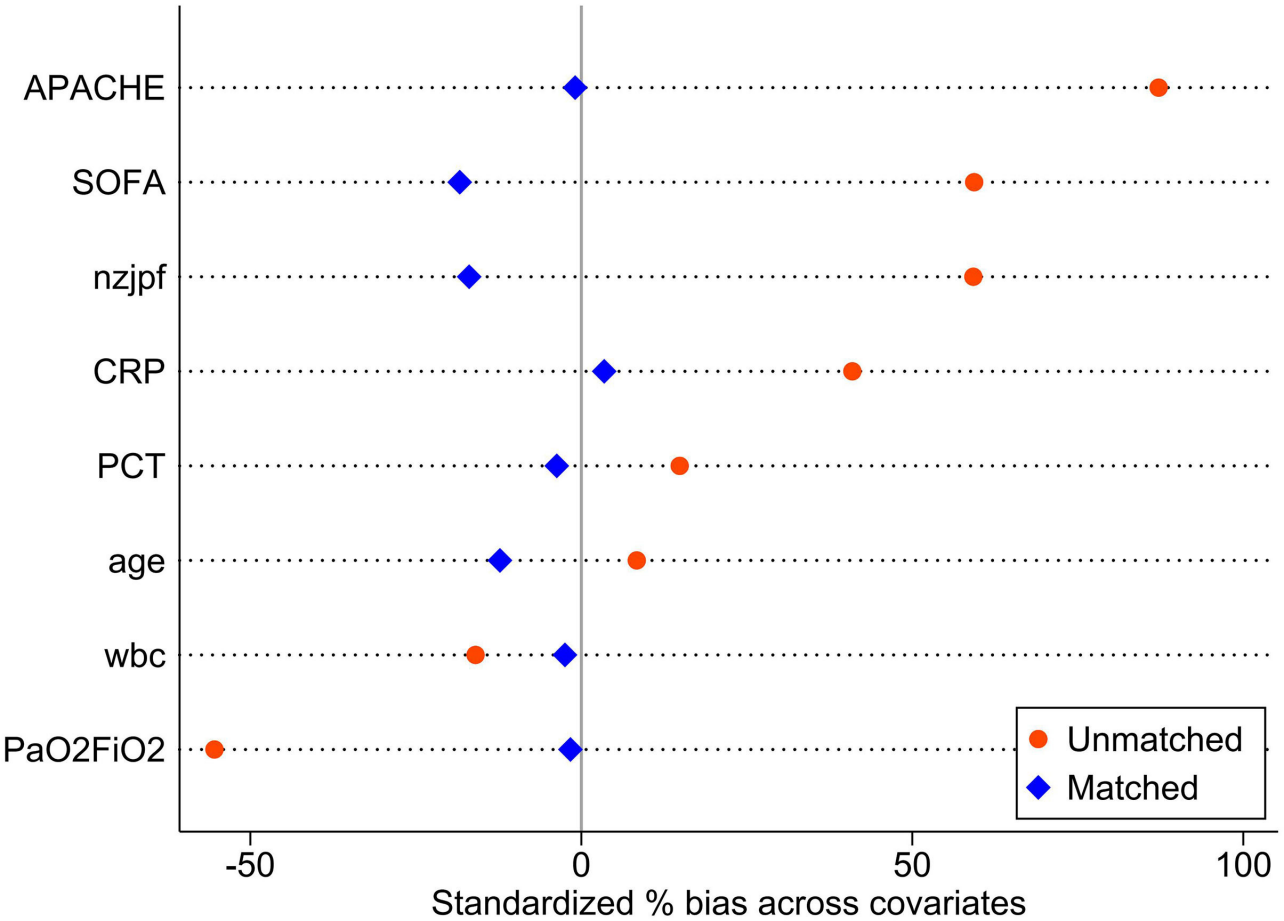
The balance of propensity score matching.

The baseline data of the two groups varied considerably; therefore, propensity matching at 1:1 was performed to reduce the heterogeneity of the two groups. The summaries of balance for unmatched and matched data are shown in the Supplementary Materials and in Figure 2. In total, 110 patients were successfully matched from the two groups (*n* = 55 patients in each group). After adjustment, there was no significant difference in baseline data between the two groups, and most of the differences in clinical data were not statistically significant (*P* < 0.05). The NAB and control groups had similar comorbidities, causes for ICU admission, signs and symptoms, PaO_2_/FiO_2_ ratios, SOFA and APACHE II scores, and laboratory examination results at admission.

### NAB Treatment

The median time from admission to initiation of NAB treatment was 9 (IQR, 6–10) days, with a total daily mean dose of 9.79 ± 1.62 mg and a medication frequency of 2 ± 0.12 times per day (Table 2). The median duration of NAB treatment was 11 (IQR, 7–6) days. The mean dosage was 4.77 ± 0.72 mg per treatment.

**Table 2:** The protocol of NAB therapy.

**Variables**	**NAB group (*n* = 55)**
The length from admission to initiation of NAB	9 (6, 10)
days (median [IQR])	
Daily dose, mg	9.79 ± 1.62
Frequency, times/day	2 ± 0.12
The duration of NAB, days (median [IQR])	11 (7–16)

### Study Outcomes

**Figure 3:**
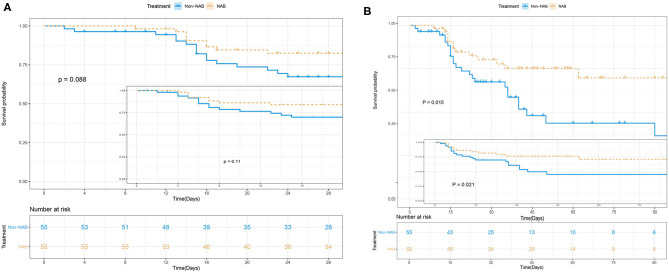
**(A)** The 28-day and **(B)** 90-day survival rate.

NAB treatment was significantly associated with decreased 90-day mortality in multivariate analyses, involving a Kaplan–Meier survival plot and a multivariable logistic regression model. The results of landmark analysis consistently revealed that NAB treatment was associated with survival benefits (Figure 3). There was a significant difference in the 90-day mortality rate between the NAB group (23.6%) and the control group (43.6%; *P* = 0.015, Figure 3B and Table 3). Five (9.1%) patients in the NAB group died during hospitalization, compared with 17 (30.9%) controls (*P* = 0.014; Table 3). At 28 days, there were 9 (16.4%) deaths in the NAB group and 16 (29.1%) in the control group (*P* = 0.088; Figure 3A).

**Table 3:** Clinical outcomes.

**During ICU stay**	**NAB group (*n* = 55)**	**No NAB group (*n* = 55)**	***P*-value**
Candida decolonization rate, n (%)	38 (69.1%)	21 (38.2%)	0.002
VAP, n (%)	6 (10.91)	9 (16.36)	0.405
Duration of mechanical ventilation, days (median [IQR])	15 (7, 29)	13 (6, 27)	0.507
Length of hospital stay, days (median[IQR])	4 0(17, 62)	27 (15, 43)	0.071
ICU length of stay, days (median[IQR])	27 (14, 53)	15 (9, 29)	0.006
Hospital mortality, n (%)	5 (9.1)	17 (30.9)	0.014
Day 28 mortality, n (%)	9 (16.4)	16 (29.1)	0.088
Day 90 mortality, n (%)	13 (23.6)	24 (43.6)	0.015
Duration of antibiotic treatment, days (median [IQR])	20 (11, 32)	16 (10, 28)	0.115

*VAP, ventilator-associated pneumonia*.

Multivariate logistic regression analysis indicated a decreased 90-day mortality in patients who received NAB (adjusted OR, 0.413; 95% CI 0.210–0.812; *P* = 0.01; Table 4). Other independent risk factors for 90-day mortality included SOFA and *Candida* scores. Similarly, the Cox's proportional hazards regression model also indicated decreased mortality with NAB treatment (adjusted HR 0.219, 95% CI 0.088–0.541, *P* = 0.001). Furthermore, to avoid selection bias, we used the IPTW method to analyze the mortality risk of patients before and after matching. The HR and 95%CI before and after matching were HR 0.96 (95% CI 0.568–1.621, *P* = 0.879) and HR 0.37(95%CI 0.152–0.715, *P* = 0.032) respectively, which was similar with the result of Cox model.

**Figure 4:**
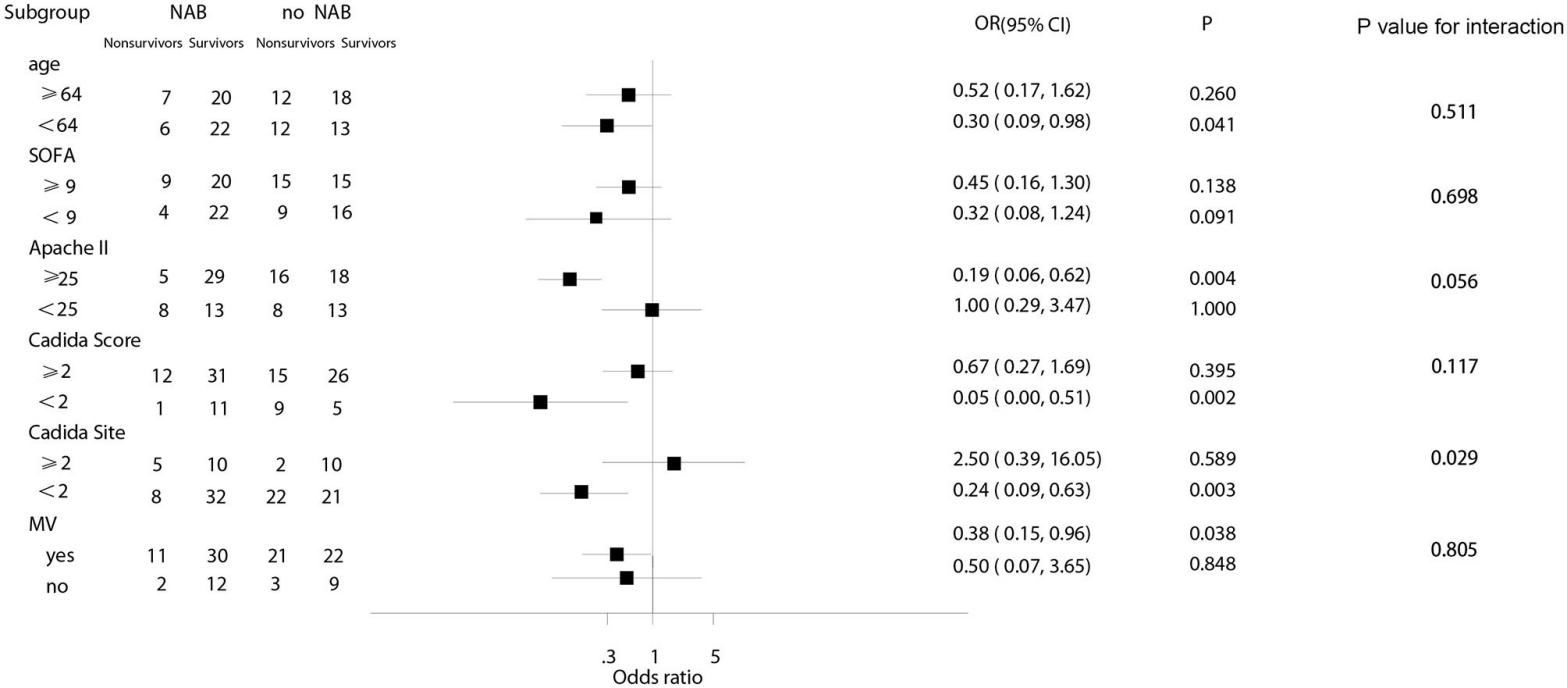
The subgroup analysis.

In subgroup analyses, the decreased risk of death at 90 days associated with NAB was consistent across subgroups for patients with a *Candida* score of 2, younger age (<64 years), a higher Apache II score (≥25), fewer *Candida* sites (<2), or MV at admission (Figure 4). Among patients with higher SOFA scores, there was no significant decrease in the risk of death (OR, 0.45; 95% CI 0.16–1.30; *P* = 0.138).

**Table 4:** Microbial results and VAP episodes.

	**NAB group (*n* = 55)**	**No NAB group (*n* = 55)**	***P*-value**
Total *Candida* spp. cultures
*Candida albicans*	35 (63.64)	32 (58.18)	0.558
Candida non-albicans			
C. tropicalis	7 (12.73)	9 (16.36)	0.589
C. paraplanatus	3 (5.45)	6 (11.11)	0.468
*C. glabrata*	16 (29.09)	14 (25.45)	1.000
*C. krusei*	1 (1.82)	1 (1.82)	1.000
Ventilator-associated pneumonia
Gram positive
*Enterococcus faecium*	1 (1.82)	2 (3.64)	1.000
*Staphylococcus*	10 (18.18)	9 (16.36)	0.801
Gram negative
A. Baumannii	25 (45.45)	16 (29.09)	0.076
*Pseudomonas aeruginosa*	6 (10.91)	14 (25.45)	0.048
*Klebsiella pneumoniae*	19(34.55)	20(36.36)	0.842
*Escherichia coli*	4 (7.27)	5 (9.09)	1.000
Antibiotic classes
Aminoglycosides	15 (27.3)	14(24.5)	0.724
Glycopeptides	25(45.5)	15(27.3)	0.074
Quinolones	15(27.3)	13(23.6)	0.603
Ureido-Penicillins	1 (1.8)	0 (0)	0.315
Carbapenems	38(69.1)	36(65.5)	0.839
Cephalosporins	25(45.5)	32(58.2)	0.252
Beta-lactamase inhibitors	34(61.8)	26(47.3)	0.180

*Candida* spp. de-colonization occurred in 38 (69.1%) patients receiving NAB and 21 (38.2%) patients in the control group (*P* = 0.002). During hospitalization, patients in the NAB group were more likely to have a longer ICU stay (median, 27 [IQR, 14–53] days) than those in the control group (15 [IQR 9–29] days; *P* = 0.006). There was no significant difference between the groups in terms of length of hospital stay, duration of MV prior to *Candida* spp. colonization, and initiation of MV at admission. The duration of MV in the ICU was 15 (IQR, 7–29) days and 13 (IQR, 6–27) days for the NAB and control groups, respectively, (*P* = 0.507; Table 3). The median time from the onset of respiratory tract *Candida* spp. colonization to initiation of MV was 6 (IQR, 4–8) and 5 (IQR, 4–7) days in the NAB and control groups, respectively; however this difference was not significant (*P* = 0.076).

### Microbial Results

In total, 312 *Candida* spp. were isolated from 275 study patients. *C. albicans* was the most frequently isolated yeast (56.7%), followed by *C. glabra*. Further, 322 bacterial strains were isolated, most of which were negative (negative bacteria, 84.4%; positive bacteria, 15.6%) in the NAB group compared with in the control group (negative bacteria, 77.6%; positive bacteria, 22.4%), but this difference was not statistically significant.

In the matched *patients, baumanii* was the most frequently isolated negative pathogen when the whole population was considered (NAB group, 45.5%; control group, 29.09%), followed by *Klebsiella pneumoniae* (34.5% and 36.36%, respectively). Bacterial resistance concerning *A. baumanii* was very serious, with an extreme drug resistance rate of 72%. Multidrug resistance accounted for 16 and 40% in the NAB and control groups, respectively; however, this between-group difference was not statistically significant (*P* = 0.759).

The distribution of *P. aeruginosa* differed between the two groups (NAB group, 10.91%; control group, 25.45%; *P* = 0.048). No significant between-group difference was found in terms of the incidence of VAP (NAB group, 10.91%; control group, 16.36%; *P* = 0.405). Carbapenems were the most commonly used antibiotics (NAB group, 69.1%; control group, 65.5%), with no significant difference between most antibiotic agents (Table 5).

**Table 5:** Multivariate logistic regression analysis for 90-mortality.

**Variables**	**HR(95% CI)**	***P*-value**
NAB treatment	0.413 (0.210, 0.812)	0.010
SOFA score	1.290 (1.19, 1.399)	0.001
APACHEII score	1.018 (0.984, 1.054)	0.293
Age	0.993 (0.976, 1.010)	0.439
PaO_2_/FiO_2_	1.00 0(0.998, 1.003)	0.732
WBC	0.952 (0.905, 1.000)	0.051
CRP	0.999(0.994,1.004)	0.656
PCT	1.011 (0.990, 1.032)	0.314
Candida score	1.088 (0.933, 1.052)	0.014

## Discussion

In this retrospective study, inhalation treatment with NAB was found to be a protective risk factor for 90-day mortality in critically ill patients with respiratory *Candida* spp. colonization who received MV for >48 h. A Cox proportional hazards regression model indicated decreased mortality with NAB treatment (adjusted HR, 0.219; 95% CI 0.088–0.541, *P* = 0.001). The *Candida* spp. de-colonization rate was 69.1% in patients receiving NAB treatment, and NAB treatment did not effectively prevent the occurrence of VAP. Notably, however, we observed a clear decrease in the number of *P. aeruginosa*-associated pneumonia episodes after *Candida* spp. de-colonization using NAB treatment. In subgroup analyses, the NAB-associated decreased risk of death at 90 days was consistent across subgroups for patients with *Candida* score of 2, younger age (<64 years), a higher Apache II score (≥25), fewer *Candida*-related sites (<2), or MV at admission.

Findings on the relationship between *Candida* spp. and bacterial VAP have been controversial in recent years. One prospective cohort study found that *Candida* spp. airway colonization was an independent risk factor for the development of *P. aeruginosa-*associated VAP (8), which was similar to findings in our study. These results indicate that the clinical interaction of *Candida* spp. colonization and VAP is complex, but also complementary and mutually uniform.

In multivariate analysis, the administration of NAB treatment was shown not only to reduce the risk of subsequent development of *P. aeruginosa*-related VAP but, more importantly, was also associated with lower in-hospital mortality and 90-day mortality rates; however, our results differ from those of a cohort study (14), which reported that *Candida* spp. airway colonization had no effect on the incidence of VAP or ICU mortality even with increased rates of *Candida* spp. de-colonization. However, in that study, the daily application of a topical paste containing polymyxin E, tobramycin, and amphotericin B for oropharyngeal de-contamination might have influenced *Candida* spp. colonization in the lower respiratory tract. Moreover, NAB treatment was not started in all patients during selective de-contamination of the digestive tract, which is likely to have resulted in selective bias. Furthermore, timing in relation to the start of treatment varied widely. Finally, the study patients recruited into that study were less severely ill. Our study did not have these limitations. To ensure consistency of baseline data between our two patient groups, each patient was strictly matched at 1:1 according to sex, age, comorbidity, and illness severity at ICU admission, and the host immune status was adjusted according to the neutrophil-to-lymphocyte ratio to exclude the influence of immune factors on our results.

Notably, we found that NAB treatment improved 90-day mortality but had no statistically significant effect on 28-day mortality. This may have been because our study patients were generally critically ill, with longer hospitalization and ICU stays, and with complex and frequently varying conditions. In these patients, long-term and consecutive survival observations can facilitate the acquisition of more clinical information and its significance compared with a transient evaluation. Second, in our study, the de-colonization of *Candida* spp. required a longer course of amphotericin atomization, and the improvement of mortality in some patients was related to *P. aeruginosa* clearance, which was possibly associated with the colonization of *Candida* spp. Therefore, we assumed that the interaction between *Candida* spp. and bacteria was long-lasting, and that quantitative changes led to qualitative changes; however, the specific mechanisms for this require further investigation in future studies.

Antifungal therapy usually requires a longer course of treatment, short-term NAB treatment is difficult to generate an effect, and the 28-day survival rate may not fully reflect the final outcome of these patients, therefore, we believe that the 90-day survival rate could reveal the true risk more accurately. The results in this study confirm our suspicion that 28-day survival rate could underestimated the risk of death in critically ill patients with candida colonization, which help us better understand the necessity for candida de-colonization.

Despite having been previously well described, bacterial and fungal species interactions, which have major environmental and medical consequences, could be greater than previously considered, ranging from cell contact and aggregation to mixed-species biofilm, environmental modifications, and alterations of the host immune response (15, 16). More importantly, interactions between fungi and bacteria can lead to increased toxin production, relevant host damage, and severe inflammation (17). One specific mechanism may involve bacteria that induce morphological changes in *Candida* (18), and *Candida* morphology and virulence have been shown to be significantly affected in the presence of *P. aeruginosa* (19). An experimental murine model has shown that the beta-glucan component of *Candida* spp. cell walls can stimulate the release of inflammatory markers and cause alveolar macrophage and neutrophil dysfunction (20). In our study, the overall occurrence of VAP was not prevented though NAB treatment; however, the number of *P. aeruginosa*-associated pneumonia episodes clearly decreased after *Candida* spp. de-colonization with NAB treatment. To exclude the influence of antibiotics, we analyzed the use of antibiotics and the influence of chronic diseases in the two groups, and these factors were not found to be statistically significant. We hypothesized that the relationship between *Candida* spp. and *P. aeruginosa* was mutual and, in terms of survival, *Candida* spp. and *P. aeruginosa* may promote and restrict each other; therefore, it is possible that yeast and bacteria and, more specifically, their interactions may have clinical implications. Further studies are needed to confirm our hypothesis concerning this delicate balance. Therefore, NAB treatment may provide an alternative treatment option for critically ill patients with *P. aeruginosa*-associated pneumonia who require MV.

One retrospective study (21) concluded that antifungal treatment was associated with a reduced risk of VAP or tracheobronchial colonization related to *P. aeruginosa*, with borderline statistical significance (OR 0.7, 95% CI 0.5–0.9; *P* = 0.046). However, the sample size was relatively small and, after 1:2 matching, a large number (10%) of immunosuppressed patients were eventually included, which may have influenced the outcome. In that study, antifungal treatment did not include inhalation of amphotericin B, which differed from our study. Furthermore, in our study, immunosuppressed patients were excluded to avoid the effect of immune status at baseline and to reduce the number of confounding factors.

In our study, *A. baumanii* was the most common pathogen, with a very high bacterial resistance rate; however, no interplay between *Candida* de-colonization and drug resistance in relation to bacterial VAP was found, which did not accord with findings reported in a study by Hamet et al. (1). In that study, *Candida* spp. airway colonization was associated with an increased risk of multidrug resistant bacterial isolation, *Candida* which could have been due to heterogeneous patient populations and possibly due to different bacterial strains, but no further analysis was undertaken to investigate this relationship in relation to *Candida* spp. de-colonization. Nevertheless, *Candida* airway colonization may be associated with increased host susceptibility to bacterial VAP, with the major pathogen being affected by antibiotic use and by other regional or epidemiologic factors.

Approximately 50% of patients in ICU are colonized with *C. albicans* (6), which was also the most common species in our study. *C. albicans* thrives in warm, moist spaces and can form hyphae and generate biofilms, which play a crucial role in the colonization of medical devices, such as urinary catheters, intravascular devices, and endotracheal tubes (22). In two previous studies (8, 23), *Candida* spp. airway colonization was reported to be associated with prolonged MV, along with prolonged hospital and ICU stay, which was similar to our study findings.

None of our study patients stopped NAB treatment because of severe adverse effects, and only a limited number of adverse effects had been documented in the medical records involving cough, bronchospasm, dyspnea, and an unpleasant aftertaste; therefore, we consider that NAB treatment is generally well tolerated and safe, which is similar to the findings of a previous study (24).

Our study had several limitations. First, this was a retrospective, observational study, which potentially limits the generalizability of our findings. Second, this study involved only a small number of patients. Had more patients been included, some of the trends observed in this study may have reached statistical significance. Third, our study was conducted in a single ICU. Therefore, our results may not be generalizable to other ICUs. Fourthly, the time span of this study was relatively large, and the changed treatment in ICU may introduce heterogeneity and bias. Finally, we recruited a population of critically ill patients; therefore, whether our results can be extrapolated to less severely ill patients is unclear.

In critically ill patients with *Candida* spp. tracheobronchial colonization who received MV for >2 days, NAB treatment was associated with a reduced risk of *P. aeruginosa*-associated VAP and improved 90-day mortality. Further prospective multicenter randomized studies are currently being undertaken to confirm our findings, and to elucidate complex yeast-bacterial interactions and their clinical significance for the human host.

## Conclusion

NAB treatment contributed to *Candida* spp. airway de-colonization was associated with a reduced risk of *P. aeruginosa* VAP, improved 90-day mortality in critically ill patients with *Candida* spp. tracheobronchial colonization who received MV for >2 days, and may be an alternative treatment option for critically ill patients with VAP.

## Data Availability Statement

The original contributions presented in the study are included in the article/Supplementary Material, further inquiries can be directed to the corresponding author/s.

## Ethics Statement

Written informed consent was not obtained from the individual(s) for the publication of any potentially identifiable images or data included in this article.

## Author Contributions

Liu J and Chen DC participated in the design of the study. Du HX, Huang BX, Wang T, Chen YZ, Liu YA and Li WZ extracted data from the information systems, Du HX and Wei LM drafted the manuscript. Statistical analysis was performed by Du HX, Ye XF, Zhang S and Huang BX. All authors read and approved the final manuscript.

## Conflict of Interest

The authors declare that the research was conducted in the absence of any commercial or financial relationships that could be construed as a potential conflict of interest.

## Publisher's Note

All claims expressed in this article are solely those of the authors and do not necessarily represent those of their affiliated organizations, or those of the publisher, the editors and the reviewers. Any product that may be evaluated in this article, or claim that may be made by its manufacturer, is not guaranteed or endorsed by the publisher.

## References

[1] Hamet M Pavon A Dalle F Pechinot A Prin S Quenot JP. *Candida* spp. airway colonization could promote antibiotic-resistant bacteria selection in patients with suspected ventilator-associated pneumonia. Intensive Care Med. (2012) 38:1272–9. 10.1007/s00134-012-2584-2 22699790

[2] He Q Wang W Zhu S Wang M Sun X. The epidemiology and clinical outcomes of ventilator-associated events among 20,769 mechanically ventilated patients at intensive care units: an observational study. Crit Care. (2021) 25:44. 10.1186/s13054-021-03484-x 33531078 PMC7851639

[3] Martin-Loeches I Torres A Povoa P Zampieri FG Rodriguez A. The association of cardiovascular failure with treatment for ventilator-associated lower respiratory tract infection. Intensive Care Med. (2019) 45:1753–62. 10.1007/s00134-019-05797-6 31620836

[4] Ziegler KM Haywood JD Sontag MK Mourani PM. Application of the New centers for disease control and prevention surveillance criteria for ventilator-associated events to a cohort of PICU patients identifies different patients compared with the previous definition and physician diagnosis. Crit Care Med. (2019) 47:e547–54. 10.1097/CCM.0000000000003766 30985451 PMC7089756

[5] Cornely OA Bassetti M Calandra T Garbino J Kullberg BJ Lortholary O. ESCMID^*^ guideline for the diagnosis and management of Candida diseases 2012: non-neutropenic adult patients. Clin Microbiol Infect. (2012) 18(Suppl. 7):19−37. 10.1111/1469-0691.12039 23137135

[6] Meersseman W Lagrou K Spriet I Maertens J Verbeken E Peetermans WE. Significance of the isolation of Candida species from airway samples in critically ill patients: a prospective, autopsy study. Intensive Care Med. (2009) 35:1526–31. 10.1007/s00134-009-1482-8 19357832

[7] Durairaj L Mohamad Z Launspach JL Ashare A Choi JY Rajagopal S. Patterns and density of early tracheal colonization in intensive care unit patients. J CritCare. (2009) 24:114–21. 10.1016/j.jcrc.2008.10.009 19272547 PMC2762407

[8] Azoulay E Timsit JF Tafflet M Lassence AD Darmon M Zahar JR. Candida colonization of the respiratory tract and subsequent pseudomonas ventilator-associated pneumonia. Chest. (2006) 129:110–7. 10.1378/chest.129.1.110 16424420

[9] Arendrup MC Chryssanthou E Gaustad P Koskela M Sandven P Fernandez V. Diagnostics of fungal infections in the Nordic countries: we still need to improve! Scand J Infect Dis. (2007) 29:S318–S. 10.1016/S0924-8579(07)71006-3 17454898

[10] Tan X Song Z Yan D Chen W Mylonakis E. *Candida* spp. airway colonization: a potential risk factor for Acinetobacter baumannii ventilator-associated pneumonia. Med Mycol. (2016) 54:myw009. 10.1093/mmy/myw009 27001670

[11] Roux D Gaudry S Dreyfuss D El-Benna J Prost ND Denamur E. *Candida albicans* impairs macrophage function and facilitates *Pseudomonas aeruginosa* pneumonia in rat. Crit Care Med. (2009) 37:1062–7. 10.1097/CCM.0b013e31819629d2 19237918

[12] Smet AD Kluytmans JA Cooper BS Mascini EM Benus RF Van TS. Decontamination of the digestive tract and oropharynx in ICU patients. N Engl J Med. (2009) 26:20–31. 10.1056/NEJMoa0800394 19118302

[13] Skrupky LP Kevin MC John D Kollef MH. A comparison of ventilator-associated pneumonia rates as identified according to the national healthcare safety network and american college of chest physicians criteria. Crit Care Med. (2011) 40:281–4. 10.1097/CCM.0b013e31822d7913 21926609

[14] Ong DS Klouwenberg P Spitoni C Bonten MJ Cremer OL. Nebulised amphotericin B to eradicate Candida colonisation from the respiratory tract in critically ill patients receiving selective digestive decontamination: a cohort study. Crit Care. (2013) 17:R233. 10.1186/cc13056 24119707 PMC4056077

[15] Peleg AY Hogan DA Mylonakis E. Medically important bacterial-fungal interactions. Nat Rev Microbiol. (2010) 8:340–9. 10.1038/nrmicro2313 20348933

[16] Arvanitis M Mylonakis E. Fungal–bacterial interactions and their relevance in health. Cell Microbiol. (2015) 17:1442–6. 10.1111/cmi.12493 26243723

[17] Morales DK Hogan DA. *Candida albicans* interactions with bacteria in the context of human health and disease. PLoS Pathogens. (2010) 6:e1000886. 10.1371/journal.ppat.1000886 20442787 PMC2861711

[18] Hogan DA. Pseudomonas-candida interactions: an ecological role for virulence factors. Science. (2002) 296:2229–32. 10.1126/science.1070784 12077418

[19] Hogan D Vik A Kolter R. A *Pseudomonas aeruginosa* quorum-sensing molecule influences *Candida albicans* morphology. Mol Microbiol. (2004) 54:1212–23. 10.1111/j.1365-2958.2004.04349.x 15554963

[20] Wimonrat Panpetch Naraporn Somboonna Dewi Embong . Oral administration of live- or heat-killed *Candida albicans* worsened cecal ligation and puncture sepsis in a murine model possibly due to an increased serum (1 → 3)-β-D-glucan. PLoS ONE. (2017) 12:e0181439. 10.1371/journal.pone.0181439 28750040 PMC5531434

[21] Nseir S Jozefowicz E Cavestri B Sendid B Pompeo CD Dewavrin F. Impact of antifungal treatment on Candida-Pseudomonas interaction: a preliminary retrospective case-control study. Intensive Care Med. (2007) 33:137–42. 10.1007/s00134-006-0422-0 17115135 PMC7095372

[22] Tan X Fuchs BB Yan W Chen W Yuen GJ Chen RB. The role of *Candida albicans* SPT20 in filamentation, biofilm formation and pathogenesis. PLos ONE. (2014) 9:e94468. 10.1371/journal.pone.0094468 24732310 PMC3986095

[23] Elebiary M Torres A Fábregas N Bellacasa JDL González J Ramirez J. Significance of the isolation of Candida species from respiratory samples in critically Ill, non-neutropenic patients. Am J Respir Crit Care Med. (1997) 156:583–90. 10.1164/ajrccm.156.2.9612023 9279244

[24] Kuiper L Ruijgrok EJ. A review on the clinical use of inhaled amphotericin B. J Aerosol Med Pulm Drug Deliv. (2009) 22:213–27. 10.1089/jamp.2008.0715 19466905

