# Supplementary material for: Effect of Nebulized Amphotericin B in Critically ill Patients With Respiratory Candida spp. De-colonization: A Retrospective Analysis
Source: Front Med (Lausanne). 2021 Sep 3;8:723904. doi: 10.3389/fmed.2021.723904 (PMC8446355; doi:10.3389/fmed.2021.723904)
Supplement: Supplementary file 1 [file Table_1.DOCX]

Supplemental materials:Patient baseline characteristics after adjustment

| Variables | NAB group (*n* = 55) | No NAB group (*n* = 55) | P Value |
| --- | --- | --- | --- |
| **Age(yr)，median(IQR)** | 63(51,74) | 64(54,76) | 0.521 |
| **Sex,male,n%** | 44(80.00) | 44(80.00) | 1 |
| **Any comorbidity，n(%)** |  |  |  |
| Chronic obstructive pulmonary disease,n (%) | 4(7.27) | 3(5.45) | 1 |
| Diabetes | 14(25.45) | 9(16.36) | 0.241 |
| Hypertension | 24(43.64) | 24(43.64) | 1 |
| Chronic cardiac disease | 12(21.82) | 13(23.64) | 0.82 |
| Chronic kidney disease | 9(16.36) | 9(16.36) | 1 |
| Chronic liver disease | 0(0.00) | 3(5.45) | 0.242 |
| Stroke | 13(23.64) | 5(9.09) | 0.059 |
| **Cause for ICU admission,n (%)** |  |  | 0.128 |
| Postoperative care | 2(3.64) | 8(14.55) |  |
| Pneumonia |  |  |  |
| HAP | 11(20.00) | 10(18.18) |  |
| CAP | 7(12.73) | 0(0.00) |  |
| AECOPD | 0(0.00) | 1(1.82) |  |
| Septic shock | 6(10.91) | 5(9.09) |  |
| Congestive heart failure | 4(7.27) | 3(5.45) |  |
| Trauma | 9(16.36) | 13(23.64) |  |
| Nerve system disease | 14(25.45) | 13(23.64) |  |
| Post-CPR | 2(3.64) | 1(1.82) |  |
| Others | 0(0.00) | 1(1.82) |  |
| **Signs and symptoms at admission,n (%)** |  |  |  |
| Fever, n (%) | 23(41.82) | 34(61.82) | 0.036 |
| Cyanosis,n (%) | 1(1.82) | 3(5.45) | 1 |
| Highest temperature (°C), median (IQR) | 37.2(36.7,37.9) | 37.6(36.9,38.4) | 0.113 |
| Systolic pressure (mmHg), median (IQR) | 130(107,160) | 128(107,146) | 0.693 |
| Diastolic pressure (mmHg), median (IQR) | 80(63,86) | 68(62,84) | 0.503 |
| Heart rate(bpm), median (IQR) | 99(82,117) | 103(85,115) | 0.600 |
| Respiratory rate (bpm), median (IQR) | 19(16,23) | 17(15,24) | 0.516 |
| Rhonchus,n (%) | 1(1.82) | 3(5.45) | 0.611 |
| Moist rales,n (%) | 12(21.82) | 14(25.45) | 1 |
| **PaO2/FiO2,median(IQR)** | 206(137,280) | 213(118,328) | 0.921 |
| **SOFA Score(IQR),median(IQR)** | 9(7,12) | 9(8,11) | 0.448 |
| **APACHE II Score(IQR)** | 26(21,31) | 25(22,29) | 0.856 |
| **Respiratory Support，n(%)** |  |  | 0.959 |
| Nasal cannula | 9(16.67) | 11(20.00) |  |
| High-flow nasal cannula | 4(7.41) | 1(1.82) |  |
| Invasive mechanical ventilation | 41(75.93) | 43(78.18) |  |
| **Physiologic parametes** |  |  |  |
| WBC(×10^9^/L), median (IQR) | 10.09(7.47,14.73) | 11.08(8.84,14.63) | 0.556 |
| Hemoglobin (g/L), median (IQR) | 101(86,115) | 107(87,125) | 0.293 |
| Platelets (×10^9^/L), median (IQR) | 148(103,224) | 134(91,191) | 0.167 |
| Lymphocytes (×10^9^/L), median (IQR) | 0.67(0.39,0.98) | 0.60(0.38,0.85) | 0.249 |
| Neutrophile granulocyte (×10^9^/L), median (IQR) | 8.46(6.00,12.99) | 10.11(7.28,12.82) | 0.390 |
| N/L Ratio, median (IQR) | 15.01(6.67,24.67) | 16.55(11.17,25.23) | 0.226 |
| PT(seconds), median (IQR) | 13.9(12.3,16.2) | 13.3(12.4,15.7) | 0.881 |
| D-dimer (μg/mL), median (IQR) | 1.93(0.72,3.74) | 2.14(0.69,5.60) | 0.584 |
| TBil (μmol/L), median (IQR) | 15.6(9.5,27.8) | 16(11.8,24.5) | 0.499 |
| Scr (μmol/L), median (IQR) | 89(59,134) | 113(74,163) | 0.231 |
| Procalcitonin (ng/mL), median (IQR) | 0.68(0.27,3.48) | 1.2(0.24,4.64) | 0.515 |
| CRP (mg/L), median (IQR) | 64.76(49,120) | 64.76(36,110) | 0.678 |
| **Number of Candida-colonized sites,median[IQR]** | 1(1,2) | 1(1,1) | 0.808 |
| **Cadida Score,median[IQR]** | 2（2，3） | 2(2,3) | 0.601 |
| **Central venous catheter,n (%)** | 33(60.0) | 41(74.5) | 0.154 |
| **Vasoactive agents,n (%)** |  |  |  |
| Norepinephrine | 33(60.0) | 35(63.6) | 0.845 |
| Epinephrine | 15(27.3) | 15(27.3) | 1.000 |
| Dopamine | 22(40) | 29(52.7） | 0.251 |
| **Ventilation from admission,days (median [IQR])** | 2（1，3） | 2（1，4） | 0.369 |
| **Duration of MV before Candida colonization ,days (median [IQR])** | 6(4,8) | 5(4,7) | 0.076 |
| **Length from admission to initiation of NAB ,days (median [IQR])** | 9(6,10) | / |  |

PT, partial thromboplastin time; TBil, total bilirubin; Scr, serum creatinine; CRP, C-reactive protein
